# *HTRA1* promoter polymorphism predisposes Japanese to age-related macular degeneration

**Published:** 2007-04-04

**Authors:** Tsunehiko Yoshida, Andrew DeWan, Hong Zhang, Ryosuke Sakamoto, Haru Okamoto, Masayoshi Minami, Minoru Obazawa, Atsushi Mizota, Minoru Tanaka, Yoshihiro Saito, Ikue Takagi, Josephine Hoh, Takeshi Iwata

**Affiliations:** 1 National Institute of Sensory Organs, National Hospital Organization Tokyo Medical Center, Tokyo, Japan; 2 Department of Epidemiology and Public Health, Yale University School of Medicine, 60 College Street, New Haven, CT; 3 Department of Ophthalmology, Juntendo University Urayasu Hospital, Chiba, Japan; 4 Division of Ophthalmology, National Hospital Organization Osaka Medical Center, Osaka, Japan; 5 Division of Ophthalmology, National Hospital Organization Kyushu Medical Center, Fukuoka, Japan

## Abstract

**Purpose:**

To study the effect of candidate single nucleotide polymorphisms (SNPs) on chromosome 10q26, recently shown to be associated with wet age-related macular degeneration (AMD) in Chinese and Caucasian cohorts, in a Japanese cohort.

**Methods:**

Using genomic DNA isolated from peripheral blood of wet AMD cases and age-matched controls, we genotyped two SNPs, rs10490924, and rs11200638, on chromosome 10q26, 6.6 kb and 512 bp upstream of the *HTRA1* gene, respectively, using temperature gradient capillary electrophoresis (TGCE) and direct sequencing. Association tests were performed for individual SNPs and jointly with SNP complement factor H (CFH) Y402H.

**Results:**

The two SNPs, rs10490924 and rs11200638, are in complete linkage disequilibrium (D'=1). Previous sequence comparisons among seventeen species revealed that the genomic region containing rs11200638 was highly conserved while the region surrounding rs10490924 was not. The allelic association test for rs11200638 yielded a p-value <10^-11^. SNP rs11200638 conferred disease risk in an autosomal recessive fashion: Odds ratio was 10.1 (95% CI 4.36, 23.06), adjusted for SNP CFH 402, for those carrying two copies of the risk allele, whereas indistinguishable from unity if carrying only one risk allele.

**Conclusions:**

The *HTRA1* promoter polymorphism, rs11200638, is a strong candidate with a functional consequence that predisposes Japanese to develop neovascular AMD.

## Introduction

Japanese patients are predominantly affected with vascular or "wet" AMD with little or no drusen deposition, in contrast to the Caucasian population which has a higher prevalence of drusen formation and the dry form of the disease. Association between the complement factor H (CFH) Y402H polymorphism (CFH 402) and age-related macular degeneration (AMD) has been shown in twelve or so different Caucasian populations [1,2]. However, that association failed to be replicated in Japanese populations, in which no control individual was found to be homozygous for the risk allele [3,4].

*HTRA1* is a member of the heat shock serine proteases and is up-regulated by cellular stress. *HTRA1* is expressed in both the human and mouse retina [5,6]. Recently a promoter single nucleotide polymorphism (SNP) rs11200638 in *HTRA1* was shown to be highly associated with wet AMD [6,7]. Furthermore, *HTRA1* resides in a region of chromosome 10q26 that has been implicated as the "top" candidate region for AMD. Here we test two SNPs, rs10490924 (6.6 kb upstream of *HTRA1*), and rs11200638, for their association to wet AMD in a Japanese population.

## Methods

We genotyped 88 neovascular AMD cases and 97 AMD-free age-matched controls for SNPs rs10490924 and rs11200638. Case and control individuals were the same as our previous CFH association study [3] with all cases being characterized as AMD grade 5B [1]. Among cases the mean age was 74.8 years (standard deviation: s.d. 8.8 years) and 70.5% male; among controls the mean age was 71.1 years (s.d. 9.1 years), and 38.1% male. Informed consent was obtained from all participants, and the procedures used conformed to the tenets of the Declaration of Helsinki. Genotyping was performed as described previously [3]. Briefly, PCR was performed using primers designed to amplify the genomic region containing each SNP (rs10490924 forward: 5'-GGT GGT TCC TGT GTC CTT CA-3', reverse: 5'-GGG GTA AGG CCT GAT CAT CT-3'; rs11200638 forward: 5'-CGG ATG CAC CAA AGA TTC TCC-3', reverse: 5'-TTC GCG TCC TTC AAA CTA ATG G-3'). Following amplification, genotype determination was performed on the PCR products using either temperature gradient capillary electrophoresis (TGCE; Reveal SpectruMedix, State College, PA) or through direct sequencing using CEQ2000XL DNA analysis system (Beckman Coulter, Fullerton, CA).

Hardy Weinberg equilibrium (HWE) χ^2^ values in the entire sample and controls only were calculated to identify possible genotyping errors. No extreme deviations (χ^2^>50) were observed (Table 1). Linkage disequilibrium (LD) was measured by the D' value. For each SNP, Pearson's χ^2^ tests with one degree of freedom for association were performed. Odds ratios (OR), population attributable risks (PAR), and their respective confidence intervals were calculated, formula in [8].

**Table 1:** Association of chromosome 10q26 single nucleotide polymorphisms with age-related macular degeneration.

**Attribute**	**rs10490924 (G/T)**	**rs11200638 (G/A)**
HWE χ^2^-combined	5.4	7.6
-controls only	0.98	0.88
Risk allele	T	A
Frequency in case	0.68	0.69
Frequency in control	0.33	0.32
Allelic association χ^2^ nominal p-value	4.74E-11	1.79E-12

To examine genotyping errors, Hardy Weinberg Equilibrium (HWE) χ^2^ values are computed with cases and controls combined and controls alone. The age range is 51 to 90 years old with mean 74.8 and standard deviation (s.d.) 8.81 in cases, and 50 to 88 years old with mean 71.1 and s.d. 9.08 in controls.

Previous functional data lead us to focus further analyses on rs11200638 [6,7]. Joint ORs for two SNPs (rs11200638 and CFH 402, previously genotyped) were calculated using standard methods [9]. Marginal ORs and their confidence intervals for the two SNP were calculated using logistic regression with SNP CFH 402 and rs11200638 as independent variables [9]. PARs were calculated using standard methods [9]. Confidence intervals around the PARs were constructed using 999 bootstrap replicates. To control for confounding, the Mantel-Hanzel test for association with two variables was used [9]. Four genotypic models were considered (Full, Recessive, Multiplicative, and Dominant) and the Aikake information criterion (AIC) was utilized to assess the fit of each model. All R scripts used in the analysis are available upon request.

## Results

SNP rs11200638, approximately 6.1 kb downstream of the surrogate SNP rs10490924, resides in the promoter of the *HTRA1* serine protease gene (512 base pairs upstream of transcriptional start site). These two SNPs were in almost complete linkage disequilibrium (LD) and showed strong association with AMD in the Hong Kong study [6] and in a Caucasian population from Utah [7].

In our cohort, the two SNPs were also in complete LD, from which only two major (frequency >5%) haplotypes, one predominant in cases and one in controls, were observed. Disease association tests yielded p-values of 4.74x10^-11^ and 1.79x10^-12^ for rs10490924 and rs11200638, respectively (Table 1). Given the previous evidence of higher conservation across species [6] and the functional consequence of rs11200638 on *HTRA1* expression [6,7], additional analyses focused on this SNP.

**Figure 1:**
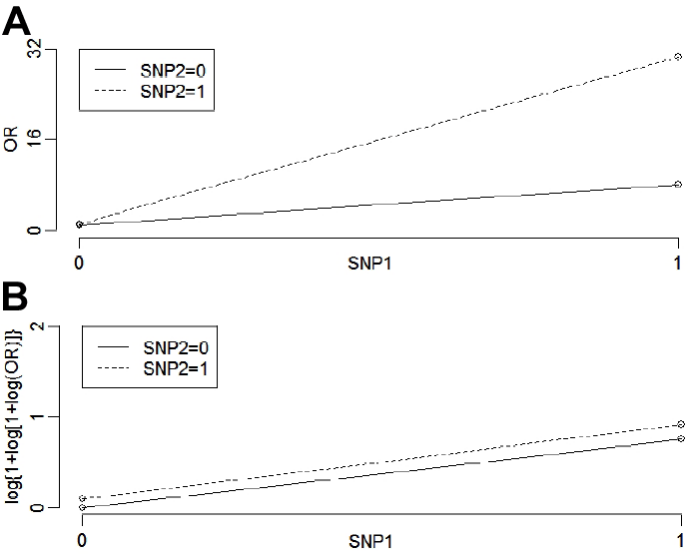
Odds ratio plots for two single nucleotide polymorphisms. Joint odds ratio plots for the single nucleotide polymorphisms (SNPs), complement factor H (CFH) 402, and rs11200638 before and after log transformation showing that the apparent interaction is a "removable" effect. SNP1=CFH 402: 0 is for TT and 1 is for CT; SNP2=rs11200638: 0 is for GG/GA and 1 is for AA. **A**: Original odds ratio (OR) curves: Height difference on the left is 1.11-1=0.11; height difference on the right is 30.52-7.92=22.60; slope for SNP2=0 is 7.92-1=6.92; slope for SNP2=1 is 30.52-1.11=29.41. **B**: Log(1+log(1+log)) transformation of the original OR.

Reanalyzing the original CFH genotype data, we found the OR covered unity (Table 2) and all interval estimates of PAR for CFH 402 variants under the four genotypic models included zero (Table 3). Of the four models, the best fit to the *HTRA1* SNP genotypic effects, as assessed by Akaike's information criterion, was the recessive model, from which the risk genotype was AA and non-risk was GG and GA (Table 3). Under the framework of recessive rs11200638 and the two observed genotypes for CFH 402, no interaction was detected between the two SNPs based on the likelihood ratio test (Table 3). Odds ratios for different genotypes of rs11200638 do not vary a great deal depending on the CFH 402 genotypes, and vice versa (Table 2). In fact, the OR curves shown in Figure 1 indicate a "removable" interaction between the two SNPs, in which the original two OR curves become parallel (i.e. no interaction after transformation with a logarithmic function). Overall, after adjusting for the CFH 402 SNP, individuals carrying the risk homozygote AA of rs11200638 are greater than 10 times more likely to have AMD than those with the other genotypes (Table 2).

**Table 2:** Odds ratios for the joint and marginal effects of single nucleotide polymorphisms complement factor H 402 and rs11200638 on age-related macular degeneration.

	**rs11200638**		
**CFH 402**	**GG/GA**	**AA**	**CFH 402 risk (adjusted for rs11200638)**
TT	1	7.92	1
CT	1.11	30.52	1.41 (95% CI: 0.54,3.74)
rs11200638 risk adjusted for CFH	1	10.02; 95% CI: 4.36,23.06	

CFH indicates complement factor H. Joint odds ratios were calculated from standard formulae. Marginal odds ratios and 95% confidence intervals were calculated using logistic regression (see Methods) with each SNP was adjusted for the other.

**Table 3:** Two-way analyses of complement factor H 402 and rs11200638.

	**PAR%**		**(95% CI)**		**M-H test: p-value**	
**Model for rs11200638**	**CFH 402**	**rs11200638**	**CFH 402**	**rs11200638**	**LRT p-value**	**AIC value**
Full	3.4 (0, 9.7)	58.3 (50.5, 64.1)	0.07	8.30E-08	0.03	221.8
Recessive	4.6 (0, 10.7)	44 (40.5, 54.0)	0.23	6.20E-09	0.12	221.5
Multiplicative	1.7 (0, 7.8)	79.8 (73.0, 88.1)	*	*	0.02	225.7
Dominant	2.2 (0, 13.7)	58.6 (43.9, 78.9)	0.91	5.80E-04	0.1	246.9

Four genotypic models for rs11200638 are considered: Let r0, r1, and r2 be the marginal relative risks for genotypes GG, GA, and AA. Then, recessive model implies r0=r1; multiplicative model implies r1=r0r2; dominant model implies r2=r1; full model does not have any restriction on relative risks except that r0, r1, r2>0. The 95% confidence intervals (CI) of population attributable risk (PAR) were obtained via a bootstrap re-sampling method with 999 replicates. Mantel-Hanzel (M-H) tests are conducted for one SNP association adjusted for the other SNP; likelihood ratio tests (LRT) for joint single nucleotide polymorphism (SNP) association under a two-way multiplicative model: the relative risk (or OR) for any genotype pair (A, B) relative to the baseline pair (A0, B0) is the product of relative risk (or OR) of A relative to A0 and that of B relative to B0. AIC denotes the Akaike's information criterion to access goodness-of-fit for the rs11200638 model.

## Discussion

These data reconfirm the association of the *HTRA1* promoter SNP rs11200638, independent of the CFH 402 polymorphism, with wet AMD. The present study genotyped two previously identified disease associated SNPs in the chromosome 10q26 region. Both SNPs showed similar significance levels. The first SNP, rs10490924, resides in the hypothetical locus, LOC387715. Several studies have found significant association between AMD and this SNP [10-12]. So far only one transcript from this hypothetical locus has been identified in one experiment. No study has identified the transcript or protein in the retina, much less identified a change in the protein as a result of the SNP. Additionally, sequence comparisons of seventeen species presented in DeWan et al. show higher sequence conservation surrounding rs11200638 compared to that around rs10490924 [6]. *HTRA1* is expressed in the retina in humans [5] and mouse [6]. Computational analysis of the *HTRA1* promoter indicate that this SNP resides in a CpG island and may result in a change in the binding site for transcription factors AP2 and SRF [6]. Preliminary functional data suggest that individuals homozygous for the risk-allele at rs11200638 exhibit increased expression of *HTRA1* [6,7]. Therefore, given the existing functional data, it appears as if the *HTRA1* promoter polymorphism, rs11200638, is likely the underlying functional polymorphism in the 10q26 region. However, the mechanism to neovascularization is yet to be understood and will require intense investigation to uncover its link to the wet form of AMD.
